# Establishment of F1 hybrid mortality in real time

**DOI:** 10.1186/s12862-017-0879-1

**Published:** 2017-01-26

**Authors:** Ashley Saulsberry, Marisa Pinchas, Aaron Noll, Jeremy A. Lynch, Seth R. Bordenstein, Robert M. Brucker

**Affiliations:** 1 Department of Biological Sciences, Vanderbilt University, Nashville, TN 37235 USA; 2 Present Address: Department of Biology, University of Utah, Salt Lake City, UT 84112 USA; 3 Present Address: Children’s Hospital Los Angeles, Los Angeles, CA 90027 USA; 4 Department of Biological Sciences, University of Illinois at Chicago, Chicago, USA; 5 Department of Pathology, Microbiology, and Immunology, Vanderbilt University, Nashville, TN 37235 USA; 6 The Rowland Institute at Harvard University, Harvard University, 100 Edwin H. Land Blvd, Cambridge, MA 02142 USA

**Keywords:** *Nasonia*, Hybrid incompatibility, Development, Reproductive isolation, Speciation

## Abstract

**Background:**

Measuring the evolutionary rate of reproductive isolation is essential to understanding how new species form. Tempo calculations typically rely on fossil records, geological events, and molecular evolution analyses. The speed at which genetically-based hybrid mortality arises, or the “incompatibility clock”, is estimated to be millions of years in various diploid organisms and is poorly understood in general. Owing to these extended timeframes, seldom do biologists observe the evolution of hybrid mortality in real time.

**Results:**

Here we report the very recent spread and fixation of complete asymmetric F_1_ hybrid mortality within eight years of laboratory maintenance in the insect model *Nasonia*. The asymmetric interspecific hybrid mortality evolved in an isogenic stock line of *N. longicornis* and occurs in crosses to *N. vitripennis* males. The resulting diploid hybrids exhibit complete failure in dorsal closure during embryogenesis.

**Conclusion:**

These results comprise a unique case whereby a strong asymmetrical isolation barrier evolved in real time. The spread of this reproductive isolation barrier notably occurred in a small laboratory stock subject to recurrent bottlenecks.

## Background

Given the importance of determining the patterns that affect the tempo of speciation, renewed emphasis has been placed on understanding how fast reproductive isolation barriers evolve during the speciation process. In the laboratory, complete F_1_ hybrid mortality has never been documented to evolve *de novo*, while there are a few cases of incomplete premating isolation [1–4] and a case of hybrid reproduction defects in experimentally evolved yeast [5]. In mammals and birds, the evolution of strong hybrid mortality takes, on average, four million and 21 million years [6–8], respectively. In frogs, estimates suggest the minimum age for total hybrid inviability to become fixed is 1.5 million years [9, 10]. However, incomplete reproductive isolation and/or segregating variation for hybrid incompatibles can occur within various species, such as *Arabidopsis* [11] and *Tribolium* [12]. Intraspecific variation in hybrid incompatibilities indicates that there is segregating variation for hybrid incompatibility alleles, but it does not inform when the incompatibility factors arose or spread per se. Taken together, the estimated timespans for severe hybrid mortality to fix in natural populations suggests that its emergence is often slow and/or restricted to large populations.

The genus *Nasonia* includes four closely related species of parasitic wasps that diverged between 0.3 to 1.0 million years ago [13]. These species include *N. vitripennis*, *N. longicornis*, *N. giraulti*, and *N. oneida*. The latter three species evolved sympatrically within the geographic range of *N. vitripennis*. The sister species, *N. giraulti* and *N. oneida*, occur sympatrically and share a distribution in the North Eastern temperate zone of North America, while *N. longicornis* lives in allopatry from *N. giraulti* and *N. oneida* on the western side of the continent. All four species are commonly used in laboratory studies of interspecific differences in their genetics, phenotypes, and microbial symbionts (reviewed in [14]).

**Fig. 1:**
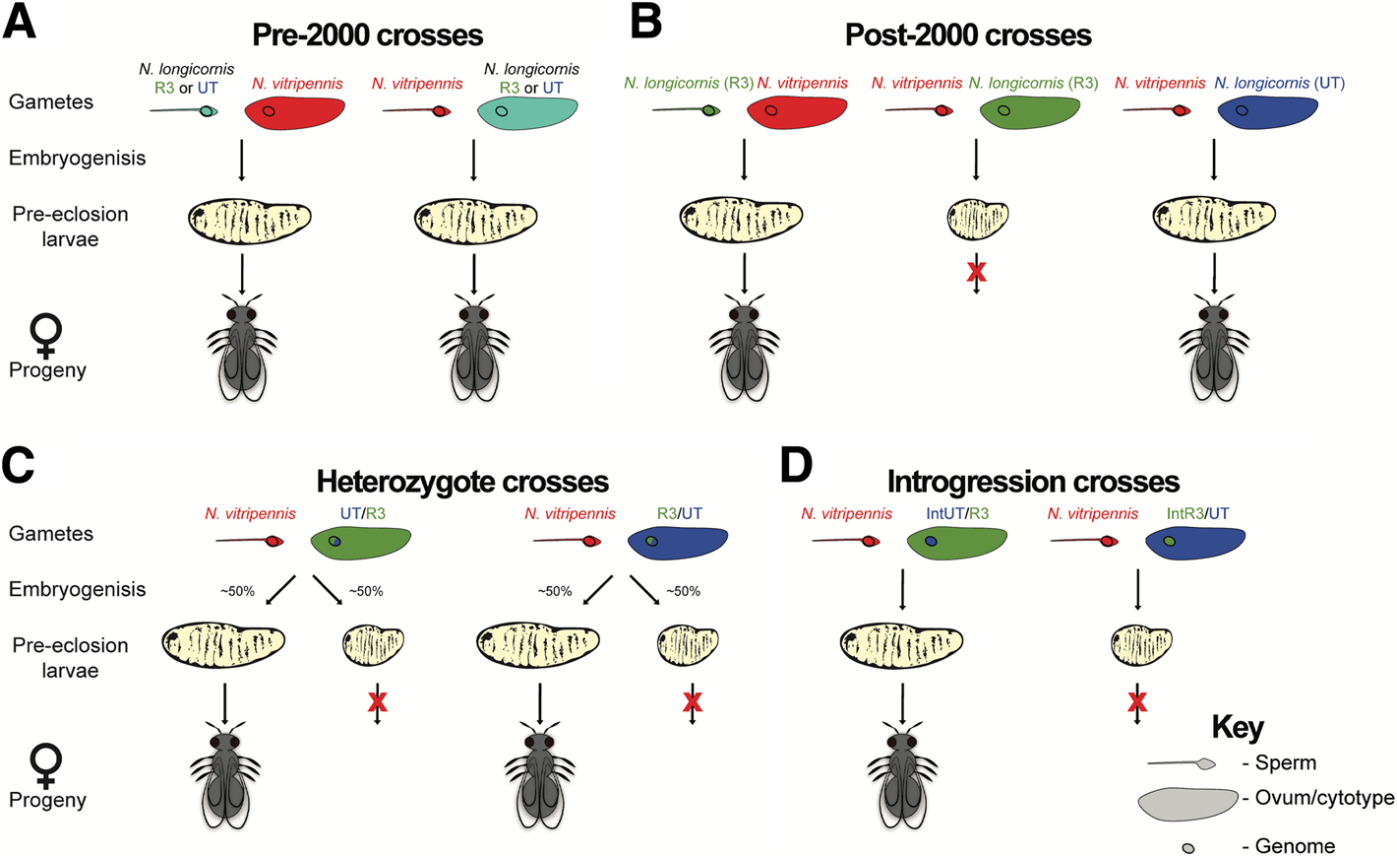
Diagram of experimental crosses and their outcomes. **a** Interspecific crosses of *Nasonia vitripennis* and *N. longicornis* strains R3 and UT produce F_1_ hybrids prior to 2008. **b** An asymmetric, postzygotic F_1_ hybrid lethality arises between *N. vitripennis* strains and *N. longicornis* strain R3 but not with *N. longicornis* strain UT. **c** To test if this asymmetric hybrid lethality is due to a maternal genetic or cytoplasmic effect, heterozygous offspring of *N. longicornis* (UT/R3 and R3/UT) were crossed with *N. vitripennis*, resulting in approximately 50% survival of F_1_ hybrid female offspring with both genotypes and cytotypes, indicating the hybrid incompatibility is not due to the R3 cytotype. **d** To further test the maternal genetic effect on hybrid mortality, *N. longicornis* strains UT and R3 were reciprocally backcrossed to each other, resulting in ~99.8% genome replacement into the alternative line’s cytotype. Females of these strains were then mated with *N. vitripennis* males, resulting in the same F_1_ hybrid lethality observed when the R3 genome was in a UT cytotype (IntR3/UT) but not when the UT genome was in the R3 cytotype (IntUT/R3)

Multiple *Wolbachia* infections exist within *Nasonia* and cause cytoplasmic incompatibility between the species [15, 16]. When reared under the same conditions in and before 2000 and cured of their *Wolbachia* infections, *N. longicornis* and *N. vitripennis* produced similar numbers of viable F_1_ hybrid offspring (90–100%) in comparison to parental controls [16] (Fig. 1a). Indeed, all intraspecific crosses in the *Nasonia* genus readily produce F_1_ hybrids in the absence of *Wolbachia* [15–17]. However, hybrid breakdown is commonly observed in the haploid F_2_ hybrid males wherein cytonuclear incompatibilities and host-microbiota interactions cause hybrid larval mortality [18–21].

Recently, while attempting to perform experiments on F_2_ hybrid breakdown between laboratory strains of *Nasonia longicornis* and *Nasonia vitripennis*, we discovered a novel F_1_ hybrid lethality between these species (Fig. 1b). It is asymmetric, complete, and occurs in the cross between *N. vitripennis* males and *N. longicornis* females. This new and unexpected F_1_ hybrid malady affords an opportunity to time the evolution of postzygotic isolation in the lab and to dissect its genetic basis. Here we describe several genetic and developmental analyses that led to timing the spread of this severe F_1_ hybrid embryonic lethality within eight years of laboratory maintenance.

## Methods

### Strains

All *Nasonia* wasps were reared in 25 °C incubators with constant light on *Sarchophaga bullata* fly pupae (‘hosts’) raised in the lab. All fly hosts were always checked for color and firmness prior to providing them to adult female *Nasonia* to ensure the quality was sufficient for parasitism. This included a dark amber to brown puparium with yellow, firm to the touch, fly pupae inside (approximately 13 days old post egg laying). Two strains of *N. longicornis* were used in this study: IV7R3-1b (R3) and NAS_NLUT230A (UT). Strain IV7R3-1b is derived from strain IV7, a *Wolbachia* infected strain that was collected in Utah and antibiotically cured of *Wobachia* in 2000 [16]. NAS_NLUT230A was collected from a natural population in Utah between 1989 and 1991, cured of *Wolbachia* by antibiotic treatment, and maintained in the laboratory according to methods described previously [22]. Two strains of *N. vitripennis* were used in this study: 13.2 and 12.1T. Strain 13.2 was derived from the R511 line collected in New York and cured of *Wolbachia* through a period of prolonged diapause in 1996 [23]. The *Wolbachia*-uninfected 12.1T strain was derived after antibiotic treatment from strain 12.1 that harbors a *Wolbachia* infection [24] and originally derived from R511 [23]. The generation time for *N. longicornis* and *N. vitripennis* under these rearing conditions is approximately two weeks.

### Collecting and mating *Nasonia*

All *Nasonia* stocks were matured into the yellow pupa stage at approximately twelve days of age and then separated according to sex to ensure virginity. After pupae eclosion, adult wasps were allowed to feed on small amounts of honey. For experimental mating crosses, one adult male and one virgin adult female *Nasonia* of the desired strains were each placed into a glass test-tube vial that was capped with a cotton plug. The pairs were observed for copulation for 5–10 min. After copulation, females were provided with honey and one to two hosts, depending on the experiment for parasitizing and then incubated under constant light at 25 °C.

### F_1_ egg, larva, and pupa counts

**Fig. 2:**
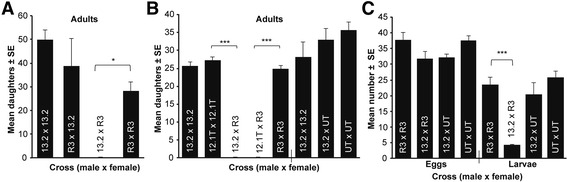
F_1_ hybrid mortality is asymmetric and embryonic. **a** Complete F_1_ hybrid reduction occurs between *N. vitripennis* males (strain 13.2) and *N. longicornis* females (strain R3). Data are represented as mean number of surviving F_1_ females (diploid) ± standard error (SE), Kolmogorov-Smirnov (K-S) test, *P < 0.05. **b** The factor responsible for complete F_1_ hybrid reduction in *N. vitripennis* is common to *N. vitripennis* strains 13.2 and 12.1T that shared an ancestor in 1996. Conversely, the factor responsible for complete F_1_ hybrid reduction in *N. longicornis* is specific to the Utah-derived strain R3, but not UT. Data are F_1_ females ± SE, K-S test, ***P < 0.0001, **c** Complete F_1_ hybrid reduction is due to hybrid mortality during embryonic development. Data are shown as mean number of F_1_ eggs and 1^st^ instar larvae ± SE, K-S test, ***P < 0.0001

Each mated female was provided honey and one unparasitized fly host with only the anterior end of the fly pupa protruding from a foam plug. This restricted the females’ ovipositing and thus localized egg laying to the anterior region of the fly pupae. Twenty-four hours later, the fly hosts were removed for offspring counts and replaced in the foam plug with new, unparasitized hosts. To characterize the F_1_ hybrid incompatibility, we reciprocally crossed *N. vitripennis* (strain 13.2) and *N. longicornis* (strain IV7R3-1b, hereafter referred to as R3) to produce F_1_ hybrids (Fig. 1b). Concurrently, we set up control self-crosses. All mating pairs are denoted as male × female. For all crosses, females were hosted once with two hosts for 24 h, and then these hosts were placed in the incubator. The *Nasonia* developed into pupae over 12–18 days before pupa counts were performed, Fig. 2a (13.2 × 13.2, *n* = 8; R3 × 13.2, *n* = 3; 13.2 × R3, *n* = 5; R3 × R3, *n* = 5). For all counts, some offspring were not counted until they had already emerged from the pupal stage to adulthood. For the experimental crosses in Fig. 2b, the females were hosted twice, and one hosting was used for pupa counts (13.2 × 13.2, *n* = 23; 12.1T × 12.1T, *n* = 13; 13.2 × R3, *n* = 23; 12.1T × R3, *n* =23; R3 × R3, *n* = 25).

To validate the strong F_1_ hybrid reduction and examine its dependency on strain background, we set up two interspecific crosses using *N. longicornis* R3 females and either *N. vitripennis* 13.2 or 12.1T males that originated from the same inbred *N. vitripennis* strain in 1996 [23] (Fig. 2b). For the crosses in Fig. 2b after the hatch mark, the females were hosted six times with the fifth set used for pupa counts (Fig. 2b, 13.2 × 13.2, *n* = 5; 13.2 × UT, *n* = 5; UT × UT, *n* = 14).

The severe reduction of F_1_ hybrids could be due to either a decrease in egg production or hybrid mortality during early developmental periods. The second set of hostings was used for egg counts to test for differences in fecundity (Fig. 2c, R3 × R3, *n* = 17; 13.2 × R3, *n* =12; 13.2 × UT, *n* = 5; UT × UT, *n* = 15). Egg counts entail carefully puncturing the fly’s puparium, removing its anterior portion, and then counting all *Nasonia* eggs observed on the fly body and in the removed section of the puparium. The third of these hostings was used for first instar larva counts to test the nature of the reduction, (Fig. 2c, R3 × R3, *n* = 18; 13.2 × R3, *n* = 10; 13.2 × UT, *n* = 6; UT × UT, *n* = 19). To calculate the number of surviving first instar larvae, the same procedure for egg counts was used except that the *Nasonia* embryos were allowed to develop undisturbed on the host for one and a half days before being counted.

**Fig. 3:**
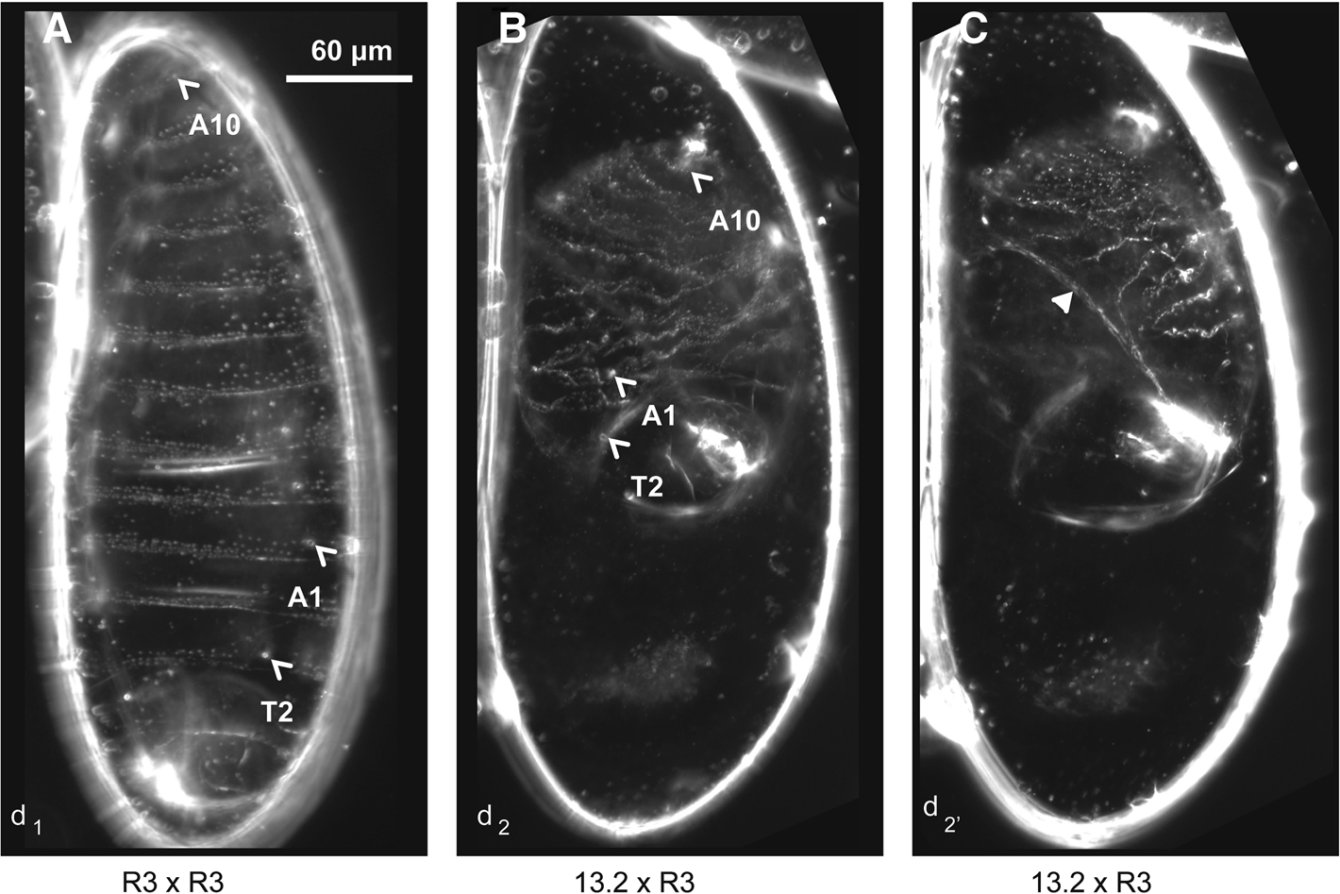
Dark field imagery of 36 h embryos. (**a**, d_1_) An R3 × R3 larval cuticle just before hatching (pre-eclosure), *T2 arrow* indicates 2^nd^ thoracic spiracle, *A1 arrow* indicates 1^st^ abdominal spiracle, *A10 arrow* indicates 10^th^ abdominal deticle belt. (**b**, d_2_) **a** representative unhatched 13.2 × R3 cuticle at two focal planes. (**c**, d_2’_) An unhatched 13.2 × R3 cuticle at two focal planes, *closed arrow* in indicates the edge of cuticle bordering the dorsal hole indicating a failure in dorsal closure

Next, we determined if the laboratory-evolved asymmetric hybrid mortality is due to a cytonuclear interaction or maternal genetic effect by generating reciprocal F_1_ heterozygous females between *N. longicornis* strains R3 and the compatible UT strain, resulting in identical nuclear genomes but cytotypes derived from either R3 or UT (Fig. 1c). For the heterozygous experimental crosses, one half of the hosts for each cross were used for egg counts the day after hosting and the other half were incubated for another 12 days and used for pupa counts. The same procedure was used for the second hosting except the hosting group halves were switched. The hostings for pupae in Fig. 3a came after the hostings for pupae in Fig. 3b (Fig. 3a, UT × UT/R3, *n* = 14; 13.2 × UT/R3, *n* = 14; 13.2 × R3/UT, *n* = 13; R3 × R3/UT, *n* = 11; Fig. 3b UT × UT/R3, *n* = 9; 13.2 × UT/R3, *n* = 12; 13.2 × R3/UT, *n* = 10; R3 × R3/UT, *n* = 9).

**Fig. 4:**
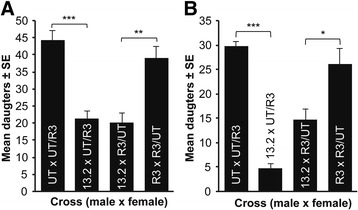
F_1_ hybrid mortality is due to a maternal genetic effect. **a** and **b** F_1_ hybrid reductions occur between *N. vitripennis* males (strain 13.2) and reciprocal *N. longicornis* herterozygous females (UT/R3 and R3/UT) for replicate experiments. Data are shown as mean number of F_1_ adult females ± SE, K-S test, *P < 0.05, **P < 0.01, and ***P < 0.0001. Crossing labels of R3/UT and UT/R3 denote heterozygous mothers derived from parents that were male/female

**Fig. 5:**
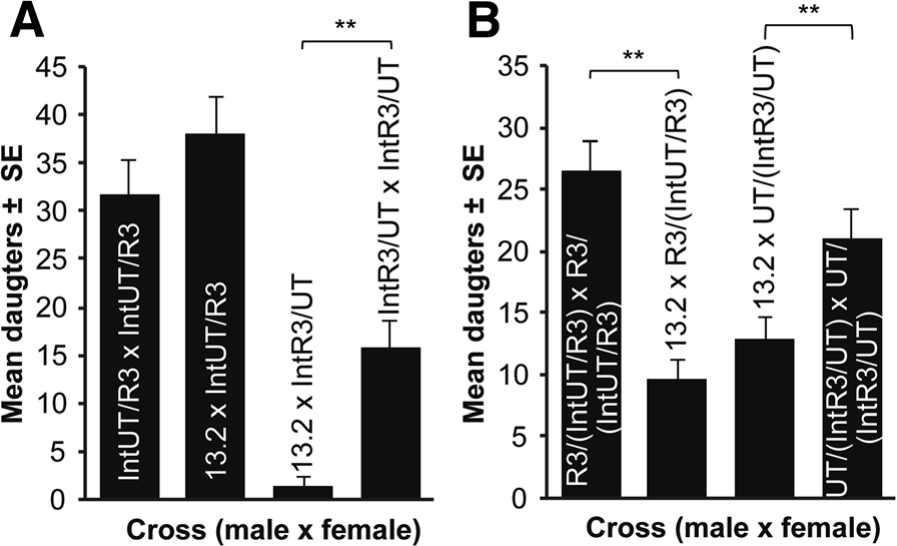
Introgression experiments corroborate a maternal genetic effect on hybrid mortality. **a** Introgression crosses. Nearly complete F_1_ hybrid mortality requires the R3 nuclear DNA, as evident by introgression of the R3 genome into the UT cytoplasm. Data are shown as mean number of adult females ± SE, K-S test, **P < 0.01. **b** Introgression-heterozygote crosses. F_1_ hybrid mortality is partial when mothers are heterozygous for the incompatible R3 and compatible UT genotypes. Data are shown as mean number of females ± SE, K-S test, **P < 0.01

To corroborate the interpretation of a maternal effect hybrid incompatibility, we generated two *N. longicornis* introgression lines by backcrossing the R3 nuclear genotype into the UT cytotype (denoted IntR3/UT) and reciprocally the UT nuclear genotype into the R3 cytotype (denoted IntUT/R3) for nine generations each (Fig. 1d). These introgression lines harbor ~99.8% of the nuclear genotype of one *N. longicornis* strain while maintaining the cytotype of the alternate strain. If the hybrid mortality is due to a maternal effect in hybrid embryos, then only the introgression line with the R3 nuclear DNA will result in hybrid death. For the introgression crosses, females were hosted six times with the fifth set used for pupa counts (Fig. 5a, IntUT/R3 × IntUT/R3, *n* = 16; 13.2 × IntUT/R3, *n* = 12; 13.2 × IntR3/UT, *n* = 7; IntR3/UT × IntR3/UT, *n* = 17). Using introgression-heterozygotes we can retest the observations of Fig. 4 but with introgressed genomes within the alternative *N. longicornis* cytotypes. For the introgression-heterozygote crosses, we generated reciprocal F_1_ heterozygotes by backcrossing the introgression lines to males of their cytotype to generate R3/(IntUT/R3) and UT/(IntR3/UT) hybrids derived from the genotypes of father/mother (Fig. 5b). Females were hosted five times with the second set of hosts used for pupa counts as previously described (Fig. 5b, R3/(IntUT/R3) × R3/(IntUT/R3), *n* = 19; 13.2 × R3/(IntUT/R3), *n* = 16; 13.2 × UT/(IntR3/UT), *n* = 21; UT/(IntR3/UT) × UT/(IntR3/UT), *n* = 20).

### Statistical analysis

Crosses that produced no offspring were excluded from the counts, likely due to poor fly host quality (average of 4.9% ± 2.1%). Crosses in which failure of fertilization occurred, as evident by all male offspring, were also excluded. Crosses that produced more than three offspring that were in diapause or were otherwise unidentifiable by sex were excluded from further analysis to avoid artifacts in the analysis because sex is unknown in the diapaused larvae. Kolmogorov-Smirnov (K-S) tests were used to compare statistical distributions of the number of daughters between each interspecific cross and corresponding intraspecific control cross for each experiment. The statistical package JMP 11 was used to perform Kolmogorov-Smirnov tests to compare egg production, female adult production, male adult production, and larva production between each interspecific cross and corresponding intraspecific control cross.

### Testing for *Wolbachia* infection

To confirm the uninfected *Wolbachia* status of the *Nasonia* strains used, DNA was extracted using the Gentra PureGene DNA extraction kit (QIAGEN®), as well as a control infected strain (*N. vitripennis* 12.1). Verification of infection status was performed using PCR with the primers ftsZuniF and fts2uniR as previously described [25]. Individual adult *Nasonia*, *n* = 4 UT, *n* = 3 R3, and *n* = 3 13.2, *n* = 3 Wolbachia positive *Nasonia* - strain 12.1, were tested and no *Wolbachia* was detected in the UT, R3, or 13.2 strains.

### Cuticle prep methods

To test embryonic and pre-eclosing larval development, eggs were collected from three R3 × R3 and three 13.2 × R3 mated females. Mated females laid eggs overnight at 25 °C in individual egg laying chambers. Eggs were collected to 1% PBS/agarose plates and incubated at 25 °C for ~48 h. Unhatched eggs and empty cuticles were counted from each type of mating. Unhatched eggs were transferred to a drop of 90:10 lactic acid:ethanol on a glass slide, covered with 22x22mm cover glass and baked at 65 °C overnight. Some R3 × R3 eggs were collected and prepared after ~30 h to catch them before hatching. Cleared cuticles were observed under dark field optics. Contrast and brightness were enhanced using Adobe Photoshop®.

## Results

All mating pairs are denoted as male × female. Figure 2a shows that while there are no significant differences in hybrids produced in the interspecific cross R3 × 13.2 and control cross 13.2 × 13.2 (Kolmogorov-Smirnov, *P* = 0.97, hereafter referred to as K-S), there is a significant and marked lack of hybrids observed in the reciprocal interspecific cross 13.2 × R3 compared to control cross R3 × R3 (K-S, *P* = 0.014). Thus, F_1_ hybrid reduction is contingent on having a 13.2 father and R3 mother. Due to the haplodiploid sex determination of *Nasonia*, F_1_ haploid sons are not hybrids, develop from unfertilized eggs, and arise from the maternal genotype. Accordingly, in all of the inter- and intraspecific crosses above and hereafter, there are no significant differences in F_1_ male survival (Fig. 2a, 13.2 × R3 compared to R3 × R3, K-S, *P* = 0.769; 13.2 × R3 and 12.1T × R3 compared to R3 × R3, K-S, *P* = 0.969 and *P* = 0.405 respectfully).

For the two interspecific crosses using *N. longicornis* R3 females and either *N. vitripennis* 13.2 or 12.1T (Fig. 2b) males, there were no hybrids produced. Conversely, the R3 × R3 control cross yielded normal numbers of offspring (Fig. 2b, K-S, *P* < 0.0001 for both comparisons). Since the underlying genetic factor for the hybrid reduction is presumably in both the *N. vitripennis* 13.2 and 12.1T strains, it is likely that the incompatibility originated prior to their 1996 splitting [23]. Next, we assessed whether the incompatibility in *N. longicornis* is strain dependent by crossing 13.2 males with females from a second *N. longicornis* strain, NAS_NLUT 230A (hereafter referred to as UT). The UT and R3 strains were collected in Utah several decades ago. Figure 2b shows that there was no reduction in F_1_ hybrids in the 13.2 × UT cross compared to the control UT × UT cross (K-S, *P* = 0.6896). Therefore, the F_1_ hybrid reduction is specific to the extant strain R3 that was previously compatible with the same *N. vitripennis* strain 13.2 in the year 2000 [16]. Likewise, when R3 was reestablished from larval diapause stocks from 2008, we again observed F_1_ hybrid reduction in the 13.2 × R3 interspecific cross compared to that of the self R3 × R3 (K-S, *P* = 0.0135). Thus, we calibrate the spread of the *N. longicornis* R3 incompatibility factor to an eight-year time span between the years 2000 and 2008.

For egg counts, we observed no significant differences in fecundity between the interspecific cross 13.2 × R3 and the control cross R3 × R3 (Fig. 2c, K-S, *P* = 0.22). Thus, the decrease in F_1_ hybrids is due to postzygotic hybrid mortality. We observed a significant 82.4% reduction in the first instar larvae in the 13.2 × R3 cross relative to the R3 × R3 control (Fig. 2c, K-S, *P* < 0.0001), indicating the hybrid mortality is primarily embryonic; the surviving larvae are haploid males. Embryonic mortality is also evident by microscopy in which 12/65 progeny from the R3 × R3 crosses failed to hatch after more than 36 h, while 66/70 from the 13.2 × R3 hybrid eggs failed to hatch (Chi-squared test, *P* < 0.0001). The hatched embryos were haploid males and not hybrids. Of the unhatched hybrid embryos, many were tiny, malformed, and clearly inviable. The cuticles of the inviable larvae had all three thoracic and ten abdominal segments, but were highly compressed (Fig. 3). Many also showed large openings on the dorsal side of the cuticle, indicating a failure in dorsal closure. Similar phenotypes have been observed in screens for embryonic lethal mutations in *Nasonia* [26] and *Drosophila* [27]. These results imply that hybrid lethality likely affects a specific developmental process, such as dorsal-ventral patterning or extraembryonic membrane specification.

The *N. longicornis* heterozygous genotypes are denoted UT/R3 and R3/UT and represent paternal/maternal origins. We crossed these heterozygous females with *N. vitripennis* 13.2 males in two replicate experiments, and observed significant hybrid reductions in crosses to both R3/UT or UT/R3 females (Fig. 4a and b). These results exclude the R3 cytotype causing hybrid mortality, and are consistent with a maternal genetic effect incompatibility between the maternal R3 genotype and 13.2 embryonic genotype, irrespective of the *N. longicornis* cytotype.

The *N. longicornis* introgression lines used to test a maternal effect on hybrid incompatibility, IntR3/UT and IntUT/R3, exhibited an asymmetric F1 hybrid lethality when crossed to *N. vitripennis* 13.2 males (Fig. 5a). Specifically, 92% hybrid mortality occurred in the incompatible cross with *N. longicornis* females containing a majority R3 genome and a UT cytoplasm (IntR3/UT). In contrast, *N. longicornis* females with the UT genome and R3 cytoplasm produced slightly more offspring (hybrids) than the control self-cross. These and the aforementioned results validate the contingency of the hybrid mortality on a R3 nuclear genotype expressed maternally during oogenesis, that in turn negatively interacts with *N. vitripennis* nuclear genes in the embryo.

We found no hybrid lethality in the crosses with heterozygous or introgression lines between the two *N. longicornis* strains, indicating that the incompatibility arose specifically between R3 and *N. vitripennis*. It is important to note that although the control IntR3/UT cross in Fig. 5a is not as fecund as the IntUT/R3 line, giving the appearance of a potential cytonuclear incompatibility between R3 genotype and UT cytotype, there is no significant difference between egg and adult production for this line (K-S, *P* = 0.675). Therefore, the incompatibility exhibited between species is not observed between strains withing the *N. longicornis* species.

In Fig. 5b, we generated reciprocal F_1_ heterozygotes using the introgression lines, R3/(IntUT/R3) and UT/(IntR3/UT); these heterozygous genotypes contain different *N. longicornis* cytotypes, but the same *N. longicornis* heterozygous nuclear genotypes. If hybrid mortality with 13.2 males is due to the proposed interaction between the R3 maternal genotype and *N. vitripennis* 13.2 embryonic genotype, then *N. longicornis* heterozygous mothers between R3 and UT should again yield partial mortality. Indeed, Fig. 5b shows that there was approximately a 50% reduction of daughters produced in both cross directions, as expected.

## Discussion

The experiments presented here demonstrate, for the first time, that complete F_1_ hybrid lethality evolved in the laboratory between *N. vitripennis* males and *N. longicornis* females between 2000 [20] to 2008. Assuming a two-week generation time, the incompatibility spread in less than 209 generations in cultures typically maintained with less than thirty foundresses per generation. The F_1_ hybrid mortality is in part due to a *N. longicornis* R3 maternal genetic effect, rather than the R3 cytotype. This conclusion is principally based on experiments crossing *N. vitripennis* males to reciprocal F_1_ heterozygous females between *N. longicornis* strains R3 and the compatible UT strain that vary in cytotype. If hybrid mortality was due to a cytonuclear interaction - which is common in F_2_ hybrid males of *Nasonia* [18–20] - then hybrid mortality would have been unidirectional and contingent on parental females harboring the R3 cytotype. Instead, we observed hybrid mortality in both crosses. Moreover, there mortality appears to involve an interaction between an embryonic product of the *N. vitripennis* genome and a maternal product of the R3 genome that is partially dominant or codominant with the maternal UT allele(s), since approximately half of the progeny of the heterozygous *N. longicornis* (UT/R3 or R3/UT) females succumb to lethality. Given that the embryo lethality seems to be related to a fairly narrow set of developmental processes (the tightly intertwined dorsoventral (DV) patterning and extraembryonic membrane specification processes) [28], a single locus developmental gene (e.g., transcription factor or signal transduction pathway component) would be a plausible genetic part of the hybrid incompatibility, though this inference awaits future experimentation.

It is possible that a polymorphism within the species of *N. longicornis* ultimately spread in the R3 line via correlated selection or drift to cause the F_1_ hybrid incompatibility, as reviewed in [29]. While selection or drift may have fixed the polymorphism from standing genetic variation in the species, the relatively few foundresses that established the R3 line and the subsequent inbreeding and bottlenecking of laboratory stocks likely reduced genetic variability and had an influence on the relatively sudden fixation within the laboratory. Previous laboratory experiments on the evolution of reproductive isolation found that selection can drive fixation of traits and reproductive barriers in a short period of time (as reviewed in [30, 31]). Experimental evolution of *Drosophila melanogaster* on a diet of EDTA led to a high degree of variability in fitness/fecundity in F_1_ hybrid crosses between the control and adapted strains [32]. Following these observations, several generations of introgression switched the third chromosome of a control strain into the background of the EDTA-adapted strain, resulting in complete sterility of females on all diets as well as lethality of both sexes at high-EDTA conditions.

While the sudden appearance of genetically-based hybrid embryonic lethality in animal hybrids is very rare, embryonic lethality in hybrid embryos occurs in other insects. For instance, several interspecific crosses within the *Drosophila* genus lead to embryonic lethality [33–35]. Similar to the incompatibility in *Nasonia*, these defects are often sex specific and depend on the direction of the cross. For example, *D. melanogaster* males × *D. simulans* females produce lethality in female embryos [34], *D. melanogaster* females × *D. santomea* males produce dead male embryos [35], and *D. montana* females × *D. texana* males leads to complete female embryo lethality in early development [33]. Unlike *Nasonia*, which lack sex chromosomes, the sex specificity in *Drosophila* seems to arise from negative interactions between the X-chromosome of one species and autosomal loci of the other. In addition, the *Drosophila* species pairs diverged 3–11 million years ago while *N. longicornis* and *N. vitripennis* diverged approximately one million years ago.

Despite these major differences between insect systems, some observations in *Drosophila* are relevant for the rapid appearance of hybrid embryonic lethality observed in *Nasonia*. A survey of *D. simulans* and *D. santomea* populations found that there is natural variation affecting the strength or presence of the hybrid embryonic lethality when crossed to *D. melanogaster* [35, 36] These and other cases *in Arabidopsis* [11] and *Tribolium* [12, 37], suggest that alleles affecting hybrid incompatibilities are segregating within populations. However, it is unclear if within-species genetic variation for hybrid incompatibilities spread recently or in the distant past. The rate at which lineages evolve intrinsic postzygotic isolation has been benchmarked against the rate of prezygotic isolation evolution [38–41]. For example, strains of allopatric *Drosophila* can have equal rates of divergence for pre- and post-zygotic reproductive isolation, while birds develop postzygotic barriers much slower [38, 41, 42]. Extremely small population sizes and relatively short time frames have also never been linked to the evolution of hybrid mortality [31]. This lack of knowledge on the tempo of evolutionary genetic changes affecting hybrid incompatibilities suggests that the spread of alleles underlying genetically-based hybrid mortality may be unusually prolonged in time and/or restricted to large populations.

In contrast, there is some evidence for the rapid evolution of bottleneck-induced premating isolation [1, 4, 43]. Various speciation theories propose that evolution (i.e., selection and/or genetic drift) in small demes can cause the spread of a particular set of traits and genetic make-up, which may lead to strong reproductive isolation [44]. For instance, hybrid lethality between species could suddenly arise if selection or population bottlenecks fix segregating variation for hybrid lethality. In the present work, we observed the rapid evolution of a complete, asymmetric, F_1_ hybrid mortality within eight years of routine laboratory rearing. These laboratory populations are maintained as small, inbred populations where strong selection or genetic drift may have rapidly promoted the spread of allele(s) for complete F_1_ interspecific hybrid lethality.

## Conclusions

Here we have characterized the establishment of severe F1 hybrid mortality in observable time. The asymmetric incompatibility spread in less than eight years under laboratory maintenance of small populations of a strain of N. longicornis and is likely due to a negative interaction between a strain-specific maternal genetic effect and a nuclear-encoded product derived from N. vitripennis in F1 hybrid embryos. Hybrid mortality appears related to a disruption of early embryonic patterning, where maternal effects and the zygotic genome first interact.

## Abbreviations

Int: Introgression
K-S: Kolmogorov-Smirnov
R3: IV7R3-1b
UT: NAS_NLUT230A
